# Skills acquisition for novice learners after a point-of-care ultrasound course: does clinical rank matter?

**DOI:** 10.1186/s12909-018-1310-3

**Published:** 2018-08-22

**Authors:** Toru Yamada, Taro Minami, Nilam J. Soni, Eiji Hiraoka, Hiromizu Takahashi, Tomoya Okubo, Juichi Sato

**Affiliations:** 1 Department of General Medicine/Family & Community Medicine, Nagoya University Graduate School of Medicine, Nagoya, Aichi Japan; 2 Department of Internal Medicine, Tokyo Bay Urayasu Ichikawa Medical Center, Urayasu, Chiba Japan; 3 Division of Pulmonary and Sleep Medicine, Care New England Medical Group, Primary Care and Specialty Services, 111 Brewster Street, Pawtucket, Rhode Island 02860 USA; 4 Department of Medicine, the Warren Alpert Medical School of Brown University, Providence, RI USA; 5 Division of General & Hospital Medicine, University of Texas Health San Antonio, San Antonio, TX USA; 6 Department of General Medicine, Juntendo University Faculty of Medicine, Tokyo, Japan; 7 Research Division, The National Center for University Entrance Examinations, Tokyo, Japan

**Keywords:** Point-of-care ultrasound, Education, Effectiveness, Trainees, Attending physician

## Abstract

**Background:**

Few studies have compared the effectiveness of brief training courses on point-of-care ultrasound (POCUS) skill acquisition of novice attending physicians vs. trainees. The purpose of this study was to evaluate the change in POCUS image interpretation skills and confidence of novice attending physicians vs. trainees after a 1-day POCUS training course.

**Methods:**

A 1-day POCUS training course was held in March 2017 in Japan. A standardized training curriculum was developed that included online education, live lectures, and hands-on training. The pre-course assessment tools included a written examination to evaluate baseline knowledge and image interpretation skills, and a physician survey to assess confidence in performing specific ultrasound applications. The same assessment tools were administered post-course, along with a course evaluation. All learners were novices and were categorized as trainees or attending physicians. Data were analyzed using two-way analysis of variance.

**Results:**

In total, 60 learners attended the course, and 51 learners (85%) completed all tests and surveys. The 51 novice learners included 29 trainees (4 medical students, 9 PGY 1–2 residents, 16 PGY 3–5 residents) and 22 attending physicians (6 PGY 6–10 physicians, and 16 physicians PGY 11 and higher). The mean pre- and post-course test scores of novice trainees improved from 65.5 to 83.9% while novice attending physicians improved from 66.7 to 81.5% (*p* < 0.001). The post-course physician confidence scores in using ultrasound significantly increased in all skill categories for both groups. Both trainees and attending physicians demonstrated similar improvement in their post-course test scores and confidence with no statistically significant differences between the groups. The course evaluation scores for overall satisfaction and satisfaction with faculty members’ teaching skills were 4.5 and 4.6 on a 5-point scale, respectively.

**Conclusions:**

Both novice trainees and attending physicians showed similar improvement in point-of-care ultrasound image interpretation skills and confidence after a brief training course. Although separate training courses have traditionally been developed for attending physicians and trainees, novice learners of point-of-care ultrasound may acquire skills at similar rates, regardless of their ranking as an attending physician or trainee. Future studies are needed to compare the effectiveness of short training courses on image acquisition skills and determine the ideal course design.

**Electronic supplementary material:**

The online version of this article (10.1186/s12909-018-1310-3) contains supplementary material, which is available to authorized users.

## Background

Point-of-care ultrasound (POCUS) is defined as ultrasonography at the patient’s bedside that is performed in real-time by a physician caring for the patient [1]. In contrast to traditional comprehensive ultrasound examinations that involve multiple providers and steps, diagnostic POCUS examinations involve the same physician determining the need for a focused examination, acquiring and interpreting the images, and incorporating the findings into the immediate management of the patient. POCUS use has been increasing in emergency medicine, critical care medicine, and internal medicine over the past two decades [1–7].

Multiple studies have demonstrated the effectiveness of POCUS training courses on knowledge and skills acquisition by different learner groups. These courses have varied in duration, content, delivery, and target audience [7, 8, 12]. Traditionally, POCUS training courses, similar to other medical courses, have targeted specific learner groups with similar levels of clinical experience, such as medical students, residents, fellows, and attending physicians. POCUS training, however, challenges this traditional training paradigm for a couple of reasons. First, POCUS use is relatively new in patient care, and most attending physicians have not yet received any POCUS training. Second, POCUS training has not yet been universally nor uniformly incorporated into undergraduate and graduate medical education [9–11]. Thus, medical students, residents, and fellows are graduating with varying levels of POCUS experience. Therefore, given the paucity of trained physicians, most POCUS training courses are geared toward novice learners, and currently, novice learners range from new medical students to experienced attending physicians.

Even though POCUS ultrasound training courses have been proven to be effective, [7, 8, 12] the effect of the same POCUS training course on skill acquisition of novice learners with different levels of clinical experience, namely trainees versus attending physicians, has not been compared. If both novice trainees and attending physicians can acquire a similar level of POCUS knowledge and skills after participating in the same beginner course, then novice trainees and attending physicians can learn side-by-side and POCUS training expenditures can be conserved. The purpose of this study was to examine the effect of the same POCUS training course on skill acquisition and confidence of novice trainees compared to novice attending physicians.

## Methods

### Design

As a preparatory step, a 2-day train-the-trainer course was conducted in November 2016 to standardize the educational curriculum for a future POCUS training course in March 2017. The train-the-trainer course curriculum was based on the principles shown to be effective for POCUS skills training [13–15]. The train-the-trainer session was led by two expert POCUS faculty from the United States. POCUS faculty were recruited from different parts of Japan based on their prior experience in teaching ultrasound and recognition as experts at their institutions. The train-the-trainer course allowed faculty an opportunity to practice, discuss, and agree upon standard techniques to teach each POCUS examination. At the conclusion of the train-the-trainer course, each faculty member’s POCUS hands-on skills were assessed to ensure standardization of skills. From November 2016 until March 2017, faculty engaged in internet-based discussions about different POCUS applications. In March 2017, a one-day review course was held just before the actual POCUS course to ensure all faculty had a clear understanding of the teaching points and techniques for each POCUS examination.

**Fig. 1:**
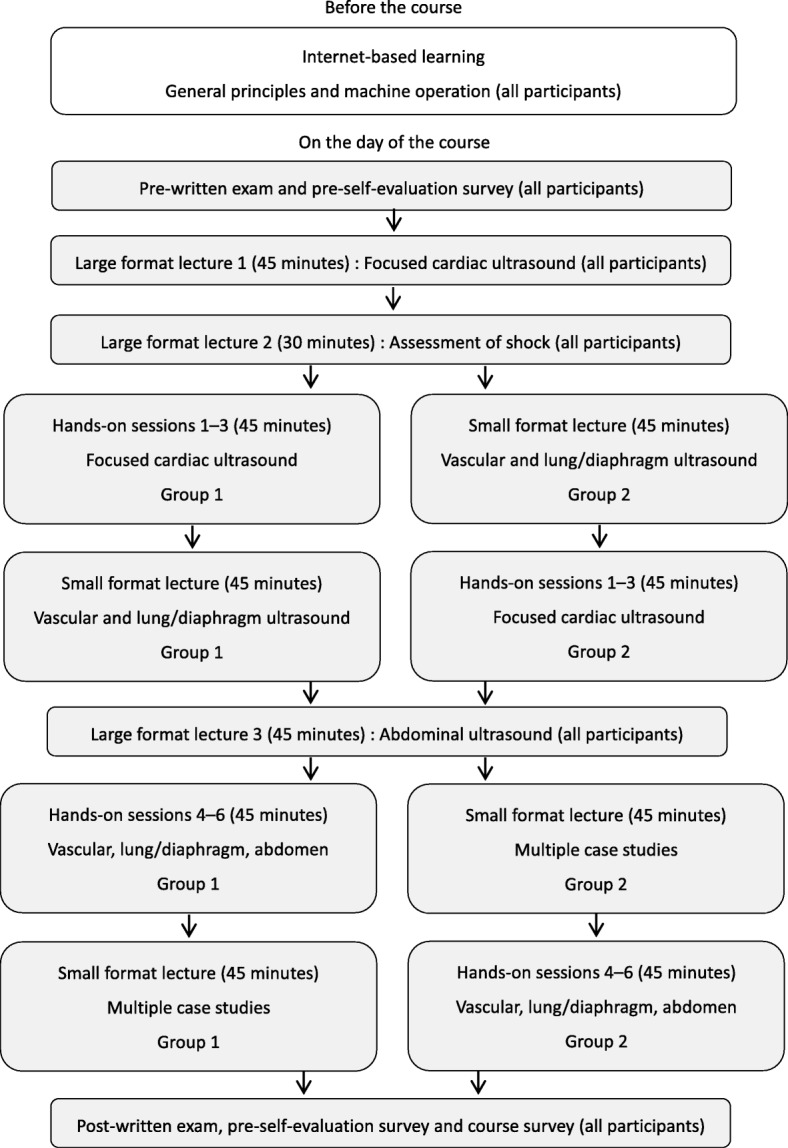
Course flow chart

The same twelve POCUS faculty that participated in the train-the-trainer course in November 2016 served as the faculty for a 1-day POCUS training course in March 2017 at the 14th Japanese Society of Hospital General Medicine Semi-Annual Meeting. The 1-day POCUS course was a separate session from the main conference and the enrollment cap was 60 participants. The 1-day POCUS course was geared toward novices learners and included pre-course internet-based modules, live lectures (3.5 h), and hands-on skills training with live models (1.5 h) (Fig. 1). Pre- and post-course written tests to evaluate learners’ POCUS knowledge and image interpretation skills were administered (Additional file 1) [8]. Learners also completed a pre- and post-course self-evaluation survey to assess their confidence in performing POCUS examinations using a five-point Likert scale (Additional file 2). Learners’ satisfaction with the training course was assessed using a post-course evaluation (Additional file 3).

The study protocol was approved by the Institutional Review Board of the Tokyo Bay Urayasu Ichikawa Medical Center. Written informed consent was obtained from all study subjects before participation.

The 1-day POCUS training course included six hands-on sessions. Each session took 15 min (1.5 h in total) and included three focused cardiac ultrasound (FOCUS) sessions, and one session each for deep vein thrombosis, lung/diaphragm, and abdominal ultrasound. The faculty-to-learner ratio was 1:3 during these hands-on sessions. Learning objectives were displayed, and faculty used standardized printed materials as teaching aids at each station (Table 1). Three course directors monitored all stations to ensure standard delivery of educational content by faculty.

**Table 1:** Educational domains of the hands-on sessions

Domains	Main learning objectives
General principles and machine operation (included in each session)	Recognition of general principles and pitfalls of ultrasound Understanding differences in probes Recognition of adequate depth, gain and common artifacts
FOCUS (Sessions 1–3)	Acquisition of PLAX, PSAX, A4C, S4C, and IVC views Interpretation of LV systolic function, pericardial effusion, and IVC
Vascular (Session 4)	Identification of examination points on the lower extremity veins Performance of compression ultrasound study of the lower extremities
Lung/diaphragm (Session 5)	Recognition of normal lung ultrasound patterns (i.e., A-lines, sliding) Recognition of diaphragm and normal diaphragm function
Abdomen (Session 6)	Identification of normal abdominal structures (i.e., kidney, gallbladder, aorta, bladder)

*Abbreviations*: *FOCUS* Focused cardiac ultrasound, *PLAX* Parasternal long-axis view, *PSAX* Parasternal short-axis, mid-ventricular level view, *A4C* Apical 4-chamber view, *S4C* Subcostal 4-chamber view, *IVC* Inferior vena cava, *LV* Left ventricular

### Subjects

Novice POCUS users were recruited to participate in the 1-day POCUS training course through marketing materials for the main conference of the Japanese Society of Hospital General Medicine. All conference attendees could participate but pre-registration was required. Subjects included senior medical students in their fifth or sixth year, residents, and attending physicians from different hospitals across Japan. All attending physicians were either trained in internal medicine or emergency medicine in Japan.

### Data collection

Learners completed a pre-course written examination and self-evaluation survey at the start and conclusion of the 1-day course. They also completed a post-course satisfaction survey prior to departing from the course. The written examination was modeled after similar examinations utilized by national professional society courses offered in the United States. All questions were presented in Japanese. The faculty teaching the hands-on skills were blinded to the written examination. Data were collected using printed materials, and answer sheets were collected at the conclusion of the pre- and post-course testing sessions.

### Definitions

We divided learners into two groups: trainees and attending physicians. In Japan, after completing medical school, graduates must participate in the two-year National Obligatory Initial Postgraduate Clinical Training Program [16, 17]. Afterwards, physicians participate in a minimum of 3 years of residency training to become board-certified in a specialty. Thus, a minimum of 5 years of postgraduate training is needed before becoming an attending physician. Therefore, we defined the trainee learner group as students and residents that were in the postgraduate years (PGY) 1–5. The attending physician learner group consisted of physicians who were PGY 6 and higher.

### Statistical analysis

Written examination and self-evaluation survey scores were analyzed with two-way analysis of variance (ANOVA). We analyzed differences in examination and survey scores between the trainees and attending physicians. Data analyses were performed using R statistical software (R version 3.1.3.)

## Results

In total, 60 learners attended the course, and 51 learners (85%) completed all tests and surveys. The 51 novice learners included 29 trainees (four medical students, nine PGY 1–2 residents, 16 PGY 3–5 residents) and 22 attending physicians (six PGY 6–10 physicians, and 16 physicians PGY11 and higher) (Table 2). All participants were novices and had never received any POCUS training.

**Table 2:** Participant characteristics (*n* = 51)

Characteristic	Total (%)	Trainees^a^ (%)	Attending physicians^b^ (%)
Number of participants	51	29	22
Sex			
Male	45 (88)	23 (79)	22 (100)
Female	6 (12)	6 (21)	0 (0)
Specialty			
Internal medicine or subspecialty	24 (47)	13 (45)	11 (50)
Critical care, emergency medicine	4 (8)	0 (0)	4 (18)
Family medicine	5 (10)	0 (0)	5 (23)
Junior resident^c^	9 (18)	9 (31)	0 (0)
Medical student^d^	4 (8)	4 (14)	0 (0)
Other	5 (10)	3 (10)	2 (9)
Setting			
University hospital	16 (31)	9 (31)	7 (32)
Community hospital	32 (63)	20 (69)	12 (54)
Private clinic	3 (6)	0 (0)	3 (14)

^a^Trainee is defined as medical students and physicians PGY 1–5

^b^Attending physician is defined as PGY 6 and higher

^c^Junior resident is defined as PGY 1 and 2 physicians who were currently in the 2-year National Obligatory Initial Postgraduate Clinical Training Program

^d^Fifth and sixth year medical students

### Image interpretation skills

**Fig. 2:**
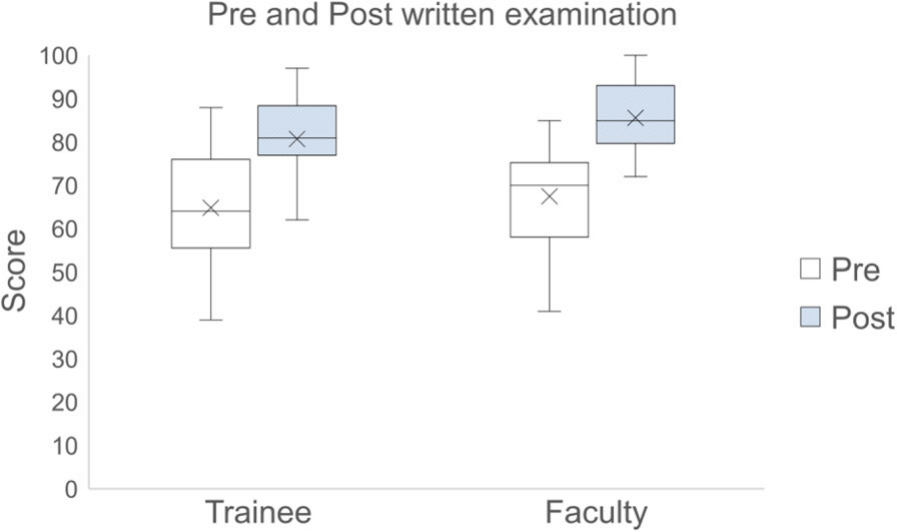
Box plot of written examination results stratified by trainee and faculty

The mean pre- and post-course written examination scores for all learners were 66.0% (standard deviation [SD] 12.9) and 82.8% (SD 9.0), respectively. The mean pre-course examination scores of trainees vs. attending physicians were 65.5% (SD 13.0) and 66.7 (SD 13.0), respectively. The mean post-course examinations scores of trainees vs. attending physicians were 83.9% (SD 9.0) and 81.5% (SD 9.0), respectively. Post-course examination scores were significantly improved compared with pre-course examination scores in both groups (*p* < 0.001). However, there was no statistically significant difference between the pre-course examination scores (*p* = 0.80) nor the post-course examination scores (*p* = 0.43) between trainees vs. attending physicians. Descriptive statistics and an ANOVA table of the examination scores for each application are shown in Additional file 4. Figure 2 shows a box plot of test results.

### Confidence scores

**Fig. 3:**
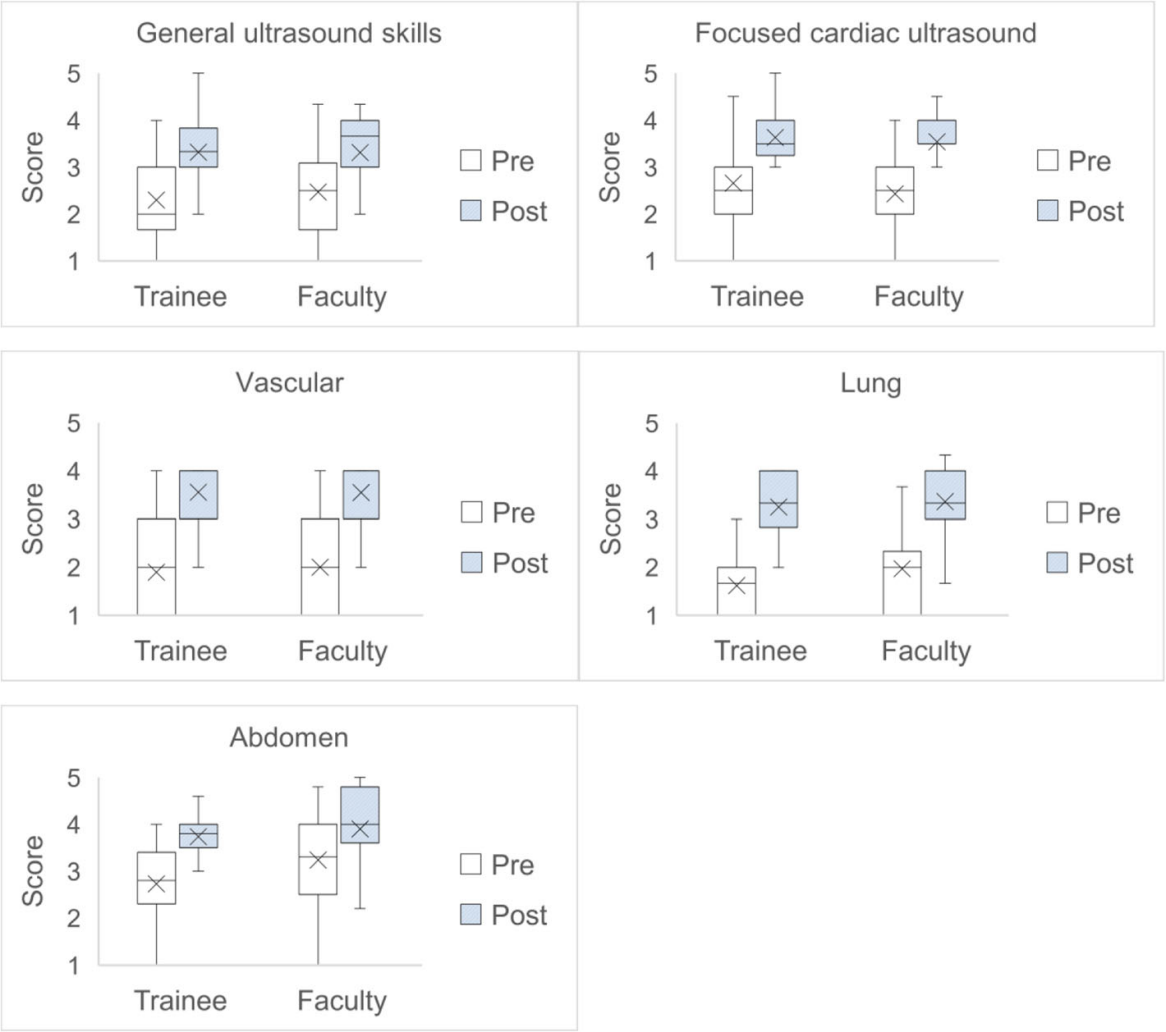
Box plot of physician survey results stratified by trainee and faculty

The mean confidence scores for all learners in the pre-course vs. post-course self-evaluation surveys were: general ultrasound skills, 2.37 (SD 0.90) vs. 3.32 (SD 0.71); focused cardiac ultrasound, 2.56 (SD 0.84) vs. 3.60 (SD 0.71); vascular diagnostics, 1.94 (SD 0.90) vs. 3.55 (SD 0.70); lung/diaphragm ultrasound, 1.77 (SD 0.76) vs. 3.30 (SD 0.70); and abdominal ultrasound, 2.95 (SD 0.97) vs 3.81 (SD 0.75). Post-course confidence scores were significantly higher compared with pre-course scores in all categories for both groups (*p* < 0.001). However, there were no significant differences in improvement of post-course confidence scores between the trainees vs. attending physicians in all categories (general ultrasound skills, *p* = 0.60; focused cardiac ultrasound, *p* = 0.68; vascular, *p* = 0.74, lung/diaphragm, *p* = 0.42; abdomen, *p* = 0.32). The pre- and post-course self-evaluation survey results are shown in Additional files 5 and 6. Figure 3 shows a box plot of self-evaluation survey results for trainees vs. attending physicians.

### Satisfaction

The course evaluation scores for overall satisfaction, satisfaction with faculty members’ teaching skills, and satisfaction with time management were 4.5, 4.6, and 3.7 on a 5-point scale, respectively.

## Discussion

Our study has three key findings. First, both novice trainees and novice attending physicians demonstrated similar improvement in POCUS knowledge, image interpretation skills, and confidence after a brief training course. Second, although shorter than other courses, a 1-day POCUS course is effective in improving POCUS skills of novice learners. Finally, we have identified a need to develop standardized POCUS training curricula for both trainees and attending physicians throughout Japan.

After physicians have access to a portable ultrasound machine, lack of training is the greatest barrier to POCUS use in clinical practice, especially for attending physicians [18–22]. However, it is unknown how to effectively train novice users of POCUS. Traditionally, POCUS training courses have been taught separately for trainees and attending physicians. But our study challenges this traditional training paradigm because both novice trainees and attending physicians had similar uptake of POCUS skills after participating in the same training course. Perhaps, categorizing POCUS learners by their baseline POCUS skill level, rather than their clinical ranking, is more important when designing POCUS training courses. Further, because integration of POCUS in undergraduate and graduate medical educational curricula has increased in recent years, [23, 24] it is possible that medical students and residents facile in POCUS could teach novice attending physicians the fundamentals of POCUS, [25] similar to peer-to-peer POCUS training that has been previously described among medical students [26–29].

The optimal duration of POCUS training courses for novice attending physicians has not been well studied. Most POCUS training courses offered by medical professional societies are typically 2 or 3 days in duration. A 3-day course offered by the American College of Chest Physicians has demonstrated improvement in POCUS knowledge, image interpretation skills, and image acquisition skills [8]. Our study is the first to demonstrate that a focused 1-day POCUS course can improve POCUS knowledge and image interpretation skills of novice attending physicians, particularly those specializing in internal medicine. For training internal medicine attending physicians, there is only one previously published study describing a 10-week institutional training program that demonstrated improvement in POCUS knowledge, image interpretation skills, and confidence.

Given the relatively low pre-course examination and confidence scores, we have identified a need to develop standardized POCUS curricula to train both trainees and attending physicians in Japan. Most primary care and subspecialty physicians in Japan have access to an ultrasound machine and can perform ultrasound examinations on their patients with minimal opposition from physicians specializing in diagnostic imaging. However, despite this favorable environment for POCUS use, no standardized POCUS training curricula have been developed for trainees or attending physicians in Japan. Therefore, to fill this important educational gap, POCUS training curricula need to be developed and integrated into undergraduate and graduate medical education for trainees, and offered as continuing medical education for attending physicians in Japan.

Our study has some limitations. We evaluated the change in learners’ POCUS image interpretation skills and confidence; however, we did not directly evaluate their image acquisition skills. Although the learners’ confidence in POCUS skills improved significantly in each category, it is not known whether or not this improvement correlates with actual improvement in image acquisition skills. Second, even though an improvement in POCUS skills was demonstrated, our sample size was small because our results are from a single training course. We recognize that our findings need to be validated using data from future courses. Additionally, a power calculation was not performed a priori, and the number of course participants was limited by the availability of instructors, equipment, and space. Last, we did not compare our 1-day POCUS training course to other courses of different lengths, such as the half-day point-of-care ultrasound pre-course offered by the Society of Hospital Medicine or the 3-day course offered by the American College of Chest Physicians [8, 30]. Therefore, although our results indicate improvement after a 1-day course, the ideal course length to maximize efficiency of POCUS skill acquisition is unknown.

## Conclusions

Both novice trainees and attending physicians showed similar improvement in point-of-care ultrasound image interpretation skills and confidence after a brief training course. Although separate training courses have traditionally been developed for attending physicians and trainees, novice learners of point-of-care ultrasound may acquire skills at similar rates, regardless of their clinical ranking as an attending physician or trainee. Future studies are needed to compare the effectiveness of short training courses on point-of-care ultrasound skills and determine the ideal course duration.

## Additional files

Additional file 1: Pre- and post-course written examinations. (DOCX 14 kb)

Additional file 2: Pre- and post-course physicians survey. (DOCX 17 kb)

Additional file 3: Post-course satisfaction surveys. (DOCX 15 kb)

Additional file 4: Descriptive statistics and an ANOVA table of the test scores for pre- and post-course written examinations. (DOCX 19 kb)

Additional file 5: Descriptive statistics for the pre- and post-course self-evaluation survey. (DOCX 19 kb)

Additional file 6: Analysis of variance table for pre- and post-course self-evaluation survey. (DOCX 22 kb)

## Abbreviations

ANOVA: Two-way analysis of variance
FOCUS: Focused cardiac ultrasound
PGY: Postgraduate year
POCUS: Point of care ultrasound
