# Supplementary material for: Skills acquisition for novice learners after a point-of-care ultrasound course: does clinical rank matter?
Source: BMC Med Educ. 2018 Aug 22;18:202. doi: 10.1186/s12909-018-1310-3 (PMC6106885; doi:10.1186/s12909-018-1310-3)
Supplement: Supplementary file 1 — Pre- and post-course written examinations. (DOCX 14 kb) [file 12909_2018_1310_MOESM1_ESM.docx]

**Additional file 1**

**Pre- and post-course written examinations**

Pre- and post-course written examinations were performed to evaluate learners’ image interpretation skills. Each examination was comprised of video images and 30 questions: 20 questions to assess simple image interpretation skills and ultrasound knowledge, and 10 questions based upon clinical scenarios to assess clinical integration of point-of-care ultrasound. The highest possible score for the pre- and post-course exams was 100 points. Questions were in multiple-choice format, with five answer choices for each question. Question domains were machine operation, focused cardiac ultrasound, vascular diagnostics, lung/diaphragm ultrasound, abdominal ultrasound, and case studies that combined these domains. Different ultrasound video images were shown for each question; however, there were an equal number of questions in each domain in the pre- and post-course exams.
