# Supplementary material for: Skills acquisition for novice learners after a point-of-care ultrasound course: does clinical rank matter?
Source: BMC Med Educ. 2018 Aug 22;18:202. doi: 10.1186/s12909-018-1310-3 (PMC6106885; doi:10.1186/s12909-018-1310-3)
Supplement: Supplementary file 2 — Pre- and post-course physicians survey. (DOCX 17 kb) [file 12909_2018_1310_MOESM2_ESM.docx]

**Additional file 2: Pre- and post-course physician survey**

| Questions | Scoring |
| --- | --- |
| General skills and machine operations | 5-agree |
| 1. I am confident in general image acquisition skills | 4-mostly agree |
| 2. I am confident in general image interpretation skills | 3-neutral |
| 3. I am confident in machine operations | 2-mostly disagree |
|  | 1-disagree |
| FOCUS |  |
| 4. I am confident in evaluation skills for cardiac systolic function |  |
| 5. I am confident in evaluation skills for inferior vena cava |  |
|  |  |
| Vascular |  |
| 6. I am confident in evaluation skills for deep vein thrombosis |  |
|  |  |
| Lung/diaphragm |  |
| 7. I am confident in evaluation skills for pneumothorax |  |
| 8. I am confident in evaluation skills for pulmonary edema |  |
| 9. I am confident in evaluation skills for diaphragmatic function |  |
|  |  |
| Abdomen |  |
| 10. I am confident in evaluation skills for cholecystitis |  |
| 11. I am confident in evaluation skills for hydronephrosis |  |
| 12. I am confident in evaluation skills for aortic aneurysm |  |
| 13. I am confident in evaluation of ureteral catheter |  |
| 14. I am confident in evaluation skills for ascites |  |

FOCUS: Focused cardiac ultrasound
