# Supplementary material for: Skills acquisition for novice learners after a point-of-care ultrasound course: does clinical rank matter?
Source: BMC Med Educ. 2018 Aug 22;18:202. doi: 10.1186/s12909-018-1310-3 (PMC6106885; doi:10.1186/s12909-018-1310-3)
Supplement: Supplementary file 3 — Post-course satisfaction surveys. (DOCX 15 kb) [file 12909_2018_1310_MOESM3_ESM.docx]

**Additional file 3: Post-course satisfaction surveys**

| Questions | Scoring |
| --- | --- |
|  |  |
| 1. Were you satisfied with this POCUS training course? | 5-extremely satisfied |
|  |  |
| 2. Were you satisfied with faculty members’ teaching skills? | 4-satisfied |
|  |  |
| 3. Were you satisfied with overall time management? | 3-neutral |
|  |  |
|  | 2-dissatisfied |
|  |  |
|  | 1-extremely dissatisfied |
|  |  |

POCUS: Point-of-care ultrasound
