# Supplementary material for: Skills acquisition for novice learners after a point-of-care ultrasound course: does clinical rank matter?
Source: BMC Med Educ. 2018 Aug 22;18:202. doi: 10.1186/s12909-018-1310-3 (PMC6106885; doi:10.1186/s12909-018-1310-3)
Supplement: Supplementary file 4 — Descriptive statistics and an ANOVA table of the test scores for pre- and post-course written examinations. (DOCX 19 kb) [file 12909_2018_1310_MOESM4_ESM.docx]

**Additional file 4: Pre- and post-course written examinations**

| Descriptive statistics of pre- and post-course written examinations | | | | |
| --- | --- | --- | --- | --- |
|  | Average pre score (SD) | | Average post score (SD) | |
| Overall (n=51) | 66.0 | (12.9) | 82.8 | (9.0) |
| Trainee (n=29) | 65.5 | (13.0) | 83.9 | (9.0) |
| Faculty (n=22) | 66.7 | (13.0) | 81.5 | (9.0) |
| SD: Standard deviation | |  |  |  |

| Analysis of variance table of pre- and post-course written examinations | | | | | |
| --- | --- | --- | --- | --- | --- |
|  | Df | SS | MS | F-value | Pr (>F) |
| TF | 1 | 8 | 8 | 0.068 | 0.795 |
| Pre-post | 1 | 7234 | 7234 | 58.144 | <0.001 |
| TF* Pre-post | 1 | 79 | 79 | 0.638 | 0.427 |
| Residuals | 98 | 12193 | 124 |  |  |
| TF: Trainee and faculty;  Df: degrees of freedom;  SS: sum of squares;  MS: mean square;  F-value: fitted value;  Pr: probability | |  |  |  |  |
