# Supplementary material for: Skills acquisition for novice learners after a point-of-care ultrasound course: does clinical rank matter?
Source: BMC Med Educ. 2018 Aug 22;18:202. doi: 10.1186/s12909-018-1310-3 (PMC6106885; doi:10.1186/s12909-018-1310-3)
Supplement: Supplementary file 5 — Descriptive statistics for the pre- and post-course self-evaluation survey. (DOCX 19 kb) [file 12909_2018_1310_MOESM5_ESM.docx]

**Additional file 5: Descriptive statistics for the pre- and post-course self-evaluation survey**

| Pre- and post-course survey question category |  | Pre-course survey mean score | (SD) | Post-course survey mean score | (SD) |
| --- | --- | --- | --- | --- | --- |
| General ultrasound skills | Overall (n=51) | 2.37 | (0.9) | 3.32 | (0.71) |
|  | Trainee (n=29) | 2.30 | (0.86) | 3.32 | (0.65) |
|  | Faculty (n=22) | 2.47 | (0.97) | 3.32 | (0.80) |
|  |  |  |  |  |  |
| Evaluation skills for FOCUS | Overall (n=51) | 2.56 | (0.84) | 3.60 | (0.71) |
|  | Trainee (n=29) | 2.66 | (0.88) | 3.64 | (0.71) |
|  | Faculty (n=22) | 2.43 | (0.79) | 3.55 | (0.74) |
|  |  |  |  |  |  |
| Evaluation skills for vascular | Overall (n=51) | 1.94 | (0.90) | 3.55 | (0.70) |
|  | Trainee (n=29) | 1.90 | (0.94) | 3.55 | (0.63) |
|  | Faculty (n=22) | 2.00 | (0.87) | 3.55 | (0.80) |
|  |  |  |  |  |  |
| Evaluation skills for lung | Overall (n=51) | 1.77 | (0.76) | 3.30 | (0.70) |
|  | Trainee (n=29) | 1.62 | (0.68) | 3.25 | (0.74) |
|  | Faculty (n=22) | 1.97 | (0.84) | 3.36 | (0.65) |
|  |  |  |  |  |  |
| Evaluation skills for abdomen | Overall (n=51) | 2.95 | (0.97) | 3.81 | (0.75) |
|  | Trainee (n=29) | 2.73 | (0.85) | 3.74 | (0.59) |
|  | Faculty (n=22) | 3.24 | (1.06) | 3.90 | (0.93) |

FOCUS: Focused cardiac ultrasound
