# Supplementary material for: Skills acquisition for novice learners after a point-of-care ultrasound course: does clinical rank matter?
Source: BMC Med Educ. 2018 Aug 22;18:202. doi: 10.1186/s12909-018-1310-3 (PMC6106885; doi:10.1186/s12909-018-1310-3)
Supplement: Supplementary file 6 — Analysis of variance table for pre- and post-course self-evaluation survey. (DOCX 22 kb) [file 12909_2018_1310_MOESM6_ESM.docx]

**Additional file 6: Analysis of variance table for pre- and post-course self-evaluation survey**

|  | Df | SS | MS | F-value | Pr (>F) |
| --- | --- | --- | --- | --- | --- |
| General ultrasound skills |  |  |  |  |  |
| TF | 1 | 0.17 | 0.175 | 0.261 | 0.611 |
| Pre-post | 1 | 22.90 | 22.903 | 34.127 | <0.001 |
| TF* Pre-post | 1 | 0.19 | 0.190 | 0.284 | 0.595 |
| Residuals | 98 | 65.77 | 0.671 |  |  |
|  |  |  |  |  |  |
|  | Df | SS | MS | F value | Pr (>F) |
| Focused cardiac ultrasound |  |  |  |  |  |
| TF | 1 | 0.62 | 0.624 | 1.017 | 0.316 |
| Pre-post | 1 | 27.54 | 27.539 | 44.904 | <0.001 |
| TF* Pre-post | 1 | 0.11 | 0.107 | 0.175 | 0.677 |
| Residuals | 98 | 60.10 | 0.613 |  |  |
|  |  |  |  |  |  |
|  | Df | SS | MS | F value | Pr (>F) |
| Vascular |  |  |  |  |  |
| TF | 1 | 0.06 | 0.06 | 0.089 | 0.767 |
| Pre-post | 1 | 65.92 | 65.92 | 98.908 | <0.001 |
| TF* Pre-post | 1 | 0.08 | 0.08 | 0.113 | 0.738 |
| Residuals | 98 | 65.32 | 0.67 |  |  |
|  |  |  |  |  |  |
|  | Df | SS | MS | F value | Pr (>F) |
| Lung |  |  |  |  |  |
| TF | 1 | 1.32 | 1.32 | 2.490 | 0.118 |
| Pre-post | 1 | 59.65 | 59.65 | 112.317 | <0.001 |
| TF* Pre-post | 1 | 0.36 | 0.36 | 0.669 | 0.416 |
| Residuals | 98 | 52.04 | 0.53 |  |  |
|  |  |  |  |  |  |
|  | Df | SS | MS | F value | Pr (>F) |
| Abdomen |  |  |  |  |  |
| TF | 1 | 2.79 | 2.786 | 3.814 | 0.05 |
| Pre-post | 1 | 18.81 | 18.808 | 25.750 | <0.001 |
| TF* Pre-post | 1 | 0.74 | 0.737 | 1.009 | 0.318 |
| Residuals | 98 | 71.58 | 0.730 |  |  |

TF: Trainee and faculty

Df: degrees of freedom;

SS: sum of squares;

MS: mean square;

F-value: fitted value;

Pr: probability
